# Unit cost repositories for health program planning and evaluation: a report on research in practice with lessons learned

**DOI:** 10.1186/s12889-023-15964-6

**Published:** 2023-06-02

**Authors:** Lori A. Bollinger, Joseph Corlis, Regina Ombam, Steven Forsythe, Stephen C. Resch

**Affiliations:** 1 Avenir Health, Glastonbury, P.O. Box 1337, CT 06033-6337 Glastonbury, USA; 2 Center for Health Decision Science, Harvard T.H. Chan School of Public Health, MA Boston, USA; 3 USAID/KEA Mission Support for Journey to Self-Reliance, Nairobi, Kenya

**Keywords:** Costs, Health financing, Resource allocation, Knowledge, Developing countries

## Abstract

**Background:**

Most low- and middle-income countries have limited access to cost data that meets the needs of health policy-makers and researchers in health intervention areas including HIV, tuberculosis, and immunization. Unit cost repositories (UCRs)—searchable databases that systematically codify evidence from costing studies—have been developed to reduce the effort required to access and use existing costing information. These repositories serve as public resources and standard references, which can improve the consistency and quality of resource needs projections used for strategic planning and resource mobilization. UCRs also enable analysis of cost determinants and more informed imputation of missing cost data. This report examines our experiences developing and using seven UCRs (two global, five country-level) for cost projection and research purposes.

**Discussion:**

We identify advances, challenges, enablers, and lessons learned that might inform future work related to UCRs. Our lessons learned include: (1) UCRs do not replace the need for costing expertise; (2) tradeoffs are required between the degree of data complexity and the useability of the UCR; (3) streamlining data extraction makes populating the UCR with new data easier; (4) immediate reporting and planning needs often drive stakeholder interest in cost data; (5) developing and maintaining UCRs requires dedicated staff time; (6) matching decision-maker needs with appropriate cost data can be challenging; (7) UCRs must have data quality control systems; (8) data in UCRs can become obsolete; and (9) there is often a time lag between the identification of a cost and its inclusion in UCRs.

**Conclusions:**

UCRs have the potential to be a valuable public good if kept up-to-date with active quality control and adequate support available to end-users. Global UCR collaboration networks and greater control by local stakeholders over global UCRs may increase active, sustained use of global repositories and yield higher quality results for strategic planning and resource mobilization.

## Background

When conducting economic evaluations or developing resource needs projections for strategic plans or budgets, analysts often perform time-consuming, ad hoc searches for data regarding the unit costs of health interventions. While these analysts may catalogue their search methods and findings, documenting unit costs and data sources in a report or in the annexes of a strategic plan, such documentation may not be widely accessible for later use. As a result, the laborious process of locating and compiling unit cost inputs and their underlying assumptions is often repeated *de novo* for each new economic evaluation and strategic planning exercise. Repeating this process results in two problems: limited potential to leverage the results of previous research and decreased time available for assessing the quality of the data that is found.

Unit cost repositories (UCRs) are searchable databases—sometimes called “unit cost databases”—that store unit cost information related to health products and services in one place [1]. Generally, UCRs are used either for costing exercises (e.g., a user looks up values to serve as an input to a resource needs projection or budgeting tasks) or for research (e.g., a broad sample of data from a UCR is analyzed to gain generalizable insights about the cost of interventions). UCRs represent a step toward increasing the transparency and quality of health cost analysis for government policy-makers and technical experts who support health sector research, planning, and resource mobilization [2]. While publicly-available databases that systematically collect data on the cost of individual health *commodities* (e.g., pharmaceuticals, equipment) have existed for a long time and are frequently consulted to obtain disaggregated data for cost analyses [3–5], recent efforts have extended this concept to compiling unit costs for entire health *interventions* based on published and sometimes unpublished costing studies.

The rationale and methodologies for developing UCRs have already been explored in previous literature [1, 6]. The first publicly available global UCR related to HIV interventions, for example, was created by the senior author of this report in 2009 in response to requests for the unit cost data underpinning UNAIDS’ Global Resource Needs Estimates [7–10]. This initial UCR evolved to become the Global Health Cost Consortium (GHCC)’s Unit Cost Study Repository [6, 11, 12] for HIV and tuberculosis.

The objective of this paper is to examine nine lessons learned related to the development, scale-up, use, and sustainability of UCRs for policy-making and research. We draw these lessons learned from our participation in the creation, data population, adaptations, and transfer of seven UCRs: the GHCC’s Unit Cost Study Repository between 2016 and 2022; the Immunization Delivery Cost Catalogue (IDCC), a web-based global UCR focusing on worldwide vaccination costs, between 2019 and 2021 [13, 14]; and five country-level UCRs for HIV which were developed as part of projects funded by the Bill & Melinda Gates Foundation in Kenya, Malawi, Mozambique, Tanzania, and Uganda between 2013 and 2022. Our individual contributions to these UCRs are detailed in the Authors’ Information section at the end of this report.

While the scope of this research in practice report focuses on the phase of developing and adapting technologies (i.e., seven discrete UCRs) for research and policy setting, the UCRs we discuss in this report have been used for a variety of purposes, including: projecting the resource needs for national strategic plans for HIV [15] and COVID-19 vaccine delivery [16]; conducting investment cases for disease programs in specific countries [17] and for multilateral organizations [18, 19]; constructing global price tags for diseases [20, 21]; and performing economic evaluations and other research studies [22–25]. Some of these databases were created and used in concurrent costing exercises, including the Tanzania HIV Investment Case 2.0 [26] and Uganda’s National Strategic Plan for HIV, 2020/2021–2024/2025 [27]; other UCRs have enabled analyses of systematically collected costs from a wide range of settings and times, leading to an improved understanding of cost drivers and efficiencies [24, 28–30] as well as the development of methods for systematically imputing previously missing or unknown values.

## Lessons learned

### Lesson 1: UCRs do not replace the need for costing expertise

Decision-makers frequently ask analysts for “one cost” per intervention to apply for all budgeting, planning, and resource allocation purposes; however, this is rarely appropriate or possible. In Mozambique, for example, we developed and populated the UCR with a range of known unit costs for community-based HIV prevention interventions for key populations [31], but these data differed in terms of underlying assumptions, methods of costing, target populations, and implementation requirements. As we populated the UCR with cost data, we recognized that improving data availability does not automatically equip policy-makers with an understanding of diverse costing methodologies and the differing results those methodologies can produce. To address this issue, we iterated a design for Mozambique’s UCR data entry form to include fields for users to check whether unit costs include specific elements (e.g., above-site costs, non-service delivery costs) or related to implementation in a specific setting (e.g., rural vs. urban, single location vs. nationwide) to aid costing analysts in identifying potential areas requiring adjustment to adapt unit costs for a specific exercise, and areas of high uncertainty. Nevertheless, the selection of a unit cost from the UCR database for use in a costing study still requires careful consideration by analysts and UCR users so that the most appropriate cost estimates are selected to match the needs of decision-makers.

### Lesson 2: Tradeoffs are required between the degree of data complexity and the useability of the UCR

While rudimentary UCRs can be as simple as spreadsheet files containing data extracted systematically from costing studies, we developed web-based UCRs like the GHCC, the IDCC, and Mozambique’s UCR by engaging with stakeholders via interviews and iterative trainings. Using insights from these engagements, we included features in the UCRs to enhance usefulness, functionality, and ease-of-use.

For example, while early stakeholders of the GHCC’s Unit Cost Study Repository expressed a preference for detailed contextual information for each unit cost estimate (e.g., primary, facility-level data; records displayed at the cost component level), the detailed information complicated the user experience and widened the user learning curve. Based on interviews with health program planners, government representatives and costing analysts to obtain feedback on the data available (e.g., desired inputs, data visualization designs, methodological issues), the GHCC iterated versions of the Unit Cost Study Repository. Ultimately, this global UCR provided users with an easy-to-use, high-level summary of unit costs and links to primary-level datasets on the GHCC website with more detailed data extracted from sources, as well as links to each citation. Between 2016 and 2019, the UCR added advanced features (e.g., data visualizations, search function), included new health areas (e.g., social and behavior change [SBC], family planning), expanded the range of interventions included, and collaboratively defined the criteria and procedures for identifying studies and extracting information to add to the repository. As the data within the GHCC’s Unit Cost Study Repository grew, tools for filtering and searching the data became increasingly important, and visualizations helped summarize the variation in the range of estimates available from multiple studies.

### Lesson 3: Streamlining data extraction makes populating the UCR with new data easier

**Fig. 1:**
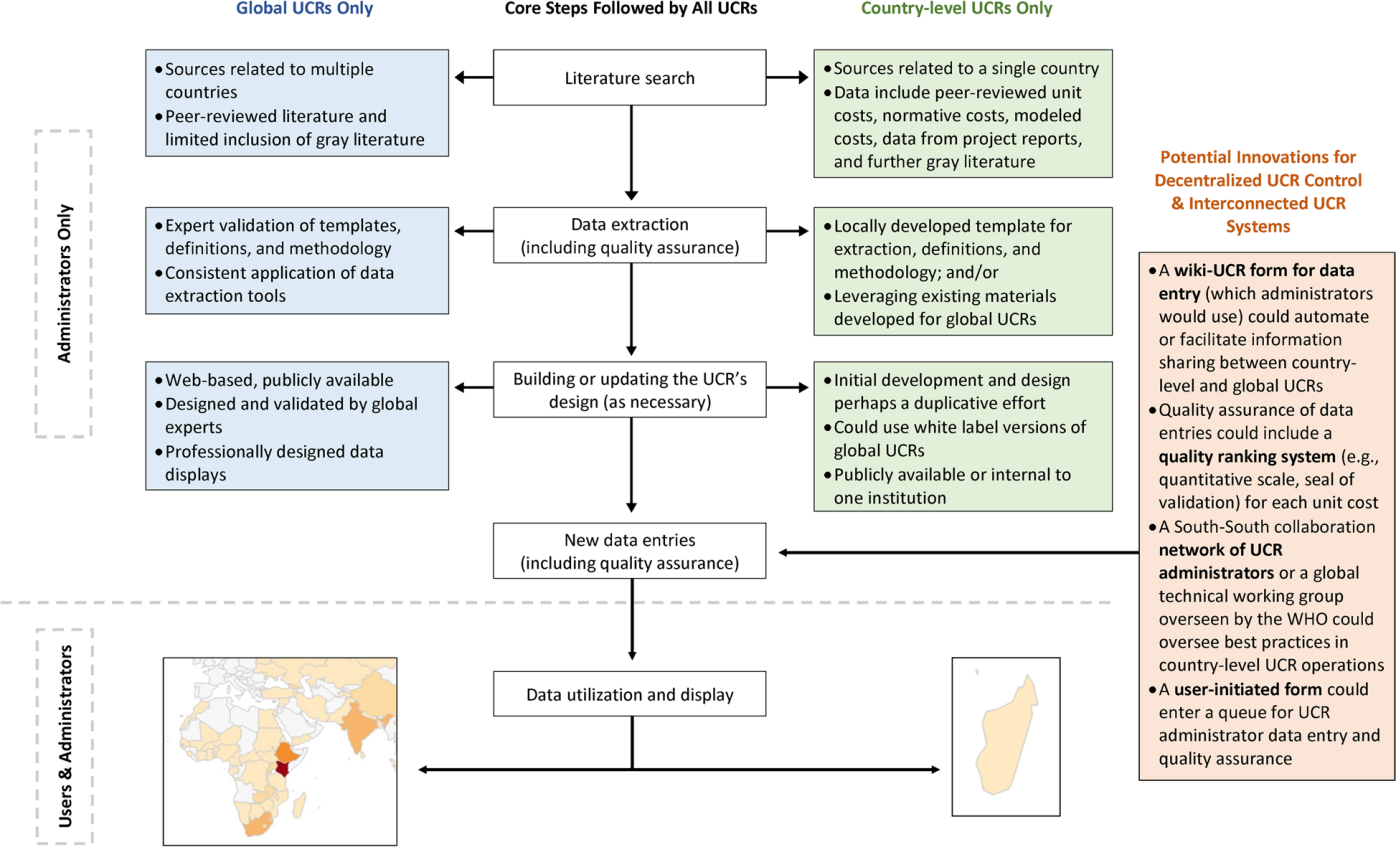
Core steps followed by UCRs and potential innovations for improved usability and sustainability. In this diagram, the boxes describe current actions and attributes for global UCRs (in blue) and for country-specific UCRs (in green). The box in orange describes potential innovations that could apply to global and country-specific UCRs. Source: The maps included in this figure are taken from the GHCC’s Unit Cost Study Repository (https://ghcosting.org/pages/data/ucsr/app/, version 6.0, updated September 30, 2021). Darker shading within the maps indicates a higher quantity of unit cost estimates included in the GHCC’s Unit Cost Study Repository. Permission to reproduce these maps in this report was granted by Avenir Health

There is a tradeoff between the amount of detail to be extracted from each source document, the effort and skill required for data extraction, and populating the database with updates (see Table 1). For example, the GHCC extracted disaggregated data at the cost component level, although these rows were collapsed in the final input files for the Unit Cost Study Repository. Based on feedback from interviews with key UCR stakeholders, the GHCC’s data extraction form was streamlined when SBC unit costs were added to the repository, removing the cost-component approach [32]. Figure 1 illustrates enablers related to data extraction, input and access, demonstrating their potential fit within the current process for developing and using UCRs.

**Table 1:** Challenges in creating, using, and sustaining UCRs with recommendations based on our lessons learned

Domain	Challenge	Implications & Recommendations
Design of UCR	The degree of data complexity available to end-users	• A high-level summary offers an entry point for novice/casual user. • Linking to and/or archiving source material satisfy users who need access to greater detail without adding complexity to data structure. • Storing and providing access to source documents within UCR (or linking to externally located source documents when direct access is not legally allowed) also creates an audit trail and increases the transparency of the extracted data.
	Data extraction	• Having a streamlined data extraction form provided users with an easier interface, possibly increasing the likelihood that people would use the UCR.
Sustainability	Short-lived interest in cost data	• IRB reviews could mandate that researchers coordinate with a UCR so that a condition for publishing articles with cost data would be uploading results to a global repository.
	Staffing requirements for UCR development and maintenance	• Donors may need to prioritize longer-term activities that promote sustained local capacity. • Absent dedicated staff, some countries are trying to task-shift current government workers (e.g., national AIDS council staff) to cover this human resource gap.
Data types and quality	Matching the needs of decision-makers with appropriate data	• The development and use of a UCR should not be viewed as the only necessary step to strengthening a costing “ecosystem” (i.e., inclusive of an organization or country’s data/tools, skills/knowledge, and processes/governance). • The selection of a unit cost requires careful consideration so that the best cost estimates can be applied to match the needs of those decision-makers.
	Tradeoffs between data quality, purpose, and amounts	• In some cases, it may be better to use cost data from a comparable setting (e.g., peer country), that it is higher quality, more recent, or better matched to the intervention being costed in the current costing exercise. • Expertise is required to appropriately identify and handle quality issues when using data from UCR in costing exercises.
	Documentation of data (i.e., sources, methodologies for determining unit costs)	• Poor documentation of methods in source material can limit the ability to identify quality issues, and ultimately diminish the usability of findings. • UCRs could include a feature for users to flag potential quality concerns.
	Data can become obsolete	• Expertise is required to know (1) whether a unit cost estimate is obsolete due to innovation in service delivery, and (2) to appropriately fill gaps in data when country-specific estimates are outdated.
Data inclusion in UCRs	Time lag in the availability of unit cost data	• Access to the most recent cost estimates should be easier for governments, donors, and researchers when global UCRs are updated regularly. • Research review boards and funders could create policies that set expectations that costing results are shared to UCRs expeditiously (e.g., within 30 days of acceptance of publication). • A global technical working group for UCRs could establish best practices for dissemination, coordinate agreements with journals so that a condition for publishing articles with cost data would be uploading complete results to one or more global repositories.
	Poor incentives for researchers to include their data in UCRs	• UCR administrators may need to implement a method of embargoing data it has obtained from authors/journals until publication date. • Potential economies of scope include an interactive data input form which could be developed for authors of costing studies to submit information to a global repository—with some additional quality control (i.e., a “Wiki-UCR”).

*AIDS *Acquired immune deficiency syndrome, *GHCC* Global Health Costing Consortium, *HIV* Human immunodeficiency virus, *IRB* Internal review board, *UCR* Unit cost repository, *VMMC* Voluntary medical male circumcision

### Lesson 4: Immediate reporting and planning needs often drive stakeholder interest in cost data

In Tanzania and Uganda, we contributed to the creation of country-level UCRs as part of the national strategic planning process. The overlap in timing was intentional in order to ensure the most up-to-date data appear in the strategy documents and then are stored in a UCR for later analysis. As we contributed to the concurrent development of the documents and the UCRs, we experienced time constraints during the time-sensitive strategic planning process. To avoid a similar situation in Mozambique, we contributed to the costing of the 5th National Strategic Plan for HIV/AIDS Response [15] by triangulating local unit costs with data for neighboring countries retrieved from GHCC’s Unit Cost Study Repository, listing the assumptions and adjustments made to the data in the strategic plan’s annexes. Once the strategic plan was ratified by the government, we built a UCR and used the data documented in the strategic plan’s annexes to populate it.

### Lesson 5: Developing and maintaining UCRs depends on dedicated staff time, which requires funding commitments

Stakeholder engagement remains perhaps the single most critical enabler for UCR development and sustainability. Bilateral and multilateral donors (e.g., USAID) and private foundations (e.g., Bill & Melinda Gates Foundation) often provide the financial resources to fund the staff hours, technology, and training costs required to create, launch, and maintain UCRs. Yet a major bottleneck for the sustainability of both global and country-level UCRs appears to be the continuation of funding—in particular, long-term funding for UCR administrators who find new data, extract details, perform quality control, and assist users with queries as necessary. Based on our experience, when investments from external sources for a UCR end and administrators no longer actively perform their role, UCR data become outdated. For example, while the GHCC’s Unit Cost Study Repository continues to see roughly the same number of new visitors each year as it did when it received funding (see Table 2), the decreased investment means that there are no administrators to update data or support users.

**Table 2:** Key metrics of the GHCC’s Unit Cost Study Repository

Funding Source	Year	Total Views^a^	Users
Bill & Melinda Gates Foundation funding for the GHCC	2018	n/a	1059
No external funding	2019	n/a	3460
	2020	n/a	2853
	2021	n/a	3587
	2022	7445	4261
	2023 (Jan-Mar)	2306	1287

^a^Total Views data began to be collected in 2022. *GHCC* Global Health Costing Consortium

Although we transferred permanent control of some UCRs to domestic government agencies (e.g., in Kenya) and national technical working groups (e.g., in Mozambique), to our knowledge, no country has established a track record of committing domestic resources to maintain or update any of the five local UCRs we helped develop.

### Lesson 6: Matching decision-maker needs with appropriate cost data can be challenging

If not a “one-stop shop” for cost data, UCRs at least serve as a “first stop” source that reduces the time analysts must spend searching for evidence. The unit cost estimates found in UCRs, however, often need to be adjusted to better match the context for which they are calculating resource requirements. For instance, during the cost analysis for the Investment Case for Jamaica (supported by SF), the country did not have access to country-specific unit cost information for post-exposure prophylaxis for HIV. Therefore, the team developing the investment case accessed the GHCC’s Unit Cost Study Repository, locating unit cost data for Suriname which was extrapolated as a best estimate [33].

When making these adjustments, analysts benefit from the information in the UCR describing the details of the original study. For example, unit costs for HIV testing programs may depend on details of the target population, location, or method of recruiting clients (e.g., mobile outreach, primary care clinics, dedicated testing centers). Moreover, as some unit cost estimates in the UCR might exclude “ingredients” such as labor, overhead or capital costs, analysts may need to impute values for their own costing exercises. Additionally, unit cost estimates might need to be adjusted for inflation or currency exchange rates. Even if a UCR is designed to indicate whether these ingredients are included or what the original study’s relevant details are, not all data entries in the UCR will rise to the same standards for documentation quality.

### Lesson 7: UCRs must have data quality control systems

Drawing from experiences developing other UCRs, we designed Mozambique’s UCR data entry form to include fields for users to check whether unit costs included specific elements (e.g., above-site costs, non-service delivery costs). Our belief was that this approach to data entry would aid costing analysts by identifying potential areas requiring adjustment to adapt unit costs for a specific exercise as well as areas of high uncertainty. Because this is a country-level UCR, local stakeholders were interested in including cost data of varying quality which had originally been generated through a range of estimation methods (e.g., modeling of costs for an intervention implemented per protocol, empirically measured cost of an intervention as implemented regardless of protocols). As noted in Lesson 2 above, there is a trade-off between including more information in a UCR and ensuring a minimum quality standard of included data. In Mozambique, we did not develop a quality assessment system that administrators can use when selecting new data for entry in the UCR (i.e., gatekeeping) as the GHCC did for its global Unit Cost Study Repository. Instead, in Mozambique, we designed the data entry form to clearly identify the type of cost data, cost ingredients, sources, links to documentation, etc. for analysts using the UCR to later refer to.

### Lesson 8: Data in UCRs can become obsolete

Once entered in UCRs, data remain accessible by users unless removed by administrators. Over time, data may become out-of-date and no longer represent current practices in a specific location or include relevant cost components (e.g., Namibia’s VMMC cost estimates in GHCC’s Unit Cost Study Repository and the country’s national strategic plan for HIV stem from a study conducted in 2010). Moreover, milestone innovations may impact the mix of interventions on offer (e.g., the introduction of Dolutegravir-based antiretroviral regimens in Mozambique altered the country’s estimated unit cost for person living with HIV on treatment per year).

While UCR administrators can conduct periodic reviews to ensure that data appearing in a repository are not obsolete, we recognize that it is not the role of UCR staff to conduct costing research to determine new unit costs for inclusion in the database.

### Lesson 9: There is often a time lag between the identification of a cost and its inclusion in UCRs

Kenya’s National AIDS Control Council proposed requiring, as part of the local IRB approval process, that cost data be shared immediately upon the completion of any costing studies and that raw data be maintained in a designated digital repository. This move was meant to fulfill four objectives: (1) reduce the time between production of new costing study results and inclusion in the UCR; (2) ensure that local researchers and policy-makers have access to primary data collected within their own country; (3) increase the value and use of the UCR for researchers and policy-makers; and (4) allay concerns of researchers about their ability to publish findings if data from their studies has already made data publicly available in UCRs.

## Conclusions

Our experience demonstrates that, while UCRs have enabled analyses of the drivers of variation in unit costs, increased access to evidence on unit costs and streamlined the process of creating resource needs projections, the databases do not represent a panacea for country costing needs. Significant expense is incurred to build, launch, and disseminate UCRs, yet these repositories stagnate without a mechanism for ongoing management, data updates, and user support.

Honest discussion of the limitations of UCRs may serve to create realistic expectations on the part of donors, local governments, and users. For example, UCRs cannot solve or compensate for an individual’s lack of expertise regarding how to generate or use cost data. Additionally, poor documentation of methods in source materials can limit a UCR administrator’s ability to control and communicate the quality of the data. Finally, when intervention implementers and evaluators have incentives to treat data from the studies they conduct as proprietary, UCRs can provide, at best, an incomplete record of relevant information.

Thus, rather than building new UCRs, stakeholders—particularly LMIC governments—may be better served by increasing investments in other areas related to unit cost data, namely: (1) routinizing systems for regularly generating and collecting cost data within national health systems, and (2) building capacity among economic researchers within the respective countries to better analyze and utilize existing cost data.

## Data Availability

The datasets used and/or analysed during the current study are available from the corresponding author on reasonable request.

## Abbreviations

COVID-19: Illness caused by Severe Acute Respiratory Syndrome Coronavirus 2
GHCC: Global Health Costing Consortium
HIV: Human immunodeficiency virus
IDCC: Immunization Delivery Cost Catalogue
LMICs: Low- and middle-income countries
SBC: Social and behavior change
UCR: Unit cost repository
UNAIDS: Joint United Nations Programme on HIV/AIDS
USAID: United States Agency for International Development
